# Maternal B Cell-Intrinsic MyD88 Signaling Mediates LPS-Driven Intrauterine Fetal Death

**DOI:** 10.3390/cells10102693

**Published:** 2021-10-08

**Authors:** Mandy Busse, Susanne Plenagl, Norina Kim Jutta Campe, Andreas J. Müller, Kerry Tedford, Anne Schumacher, Ana Claudia Zenclussen

**Affiliations:** 1 Experimental Obstetrics and Gynecology, Medical Faculty, Otto-von-Guericke University, 39108 Magdeburg, Germany; mandy.busse@med.ovgu.de (M.B.); susanne.plenagl@hotmail.de (S.P.); noricampe@googlemail.com (N.K.J.C.); 2 Institute for Molecular and Clinical Immunology, Medical Faculty, Otto-von-Guericke University, 39120 Magdeburg, Germany; andreas.mueller@med.ovgu.de; 3 Helmholtz Centre for Infection Research, 38124 Braunschweig, Germany; 4 Institute of Biochemistry and Cell Biology, Medical Faculty, Otto-von-Guericke University, 39120 Magdeburg, Germany; kerry.tedford@med.ovgu.de; 5 Department of Environmental Immunology, Helmholtz Centre for Environmental Research-UFZ, 04318 Leipzig, Germany; anne.schumacher@ufz.de; 6 Saxonian Incubator for Translation Research, Leipzig University, 04103 Leipzig, Germany

**Keywords:** intrauterine fetal death, pregnancy, inflammation, B cells

## Abstract

Immunological networks balance tolerance towards paternal alloantigens during pregnancy with normal immune response to pathogens. Subclinical infections can impact this balance and lead to preterm birth or even intrauterine fetal death (IUFD). We recently showed that loss of maternal B cells renders murine fetuses susceptible to IUFD after LPS exposure. Since the signaling pathway involved in this B-cell mediated response remains unclear, we aimed to understand the participation of MyD88 in this response using B-cell-specific MyD88-deficient (BMyD88^-/-^) mice. B cells isolated from wild-type (WT), BMyD88^-/-^, CD19^-/-^ and MyD88^-/-^ dams on gestational day (gd) 10 responded differently to LPS concerning cytokine secretion. In vivo LPS challenge on gd 10 provoked IUFD in CD19^-/-^ mothers with functional MyD88, while fetuses from BMyD88^-/-^ and MyD88^-/-^ mice were protected. These outcomes were associated with altered cytokine levels in the maternal serum and changes in CD4^+^ T-cell responses. Overall, the loss of MyD88 signaling in maternal B cells prevents the activation of cytokine release that leads to IUFD. Thus, while MyD88 signaling in maternal B cells protects the mother from infection, it ultimately kills the fetus. Understanding the cellular mechanisms underlying infection-driven pregnancy complications is the first step to designing powerful therapeutic strategies in the future.

## 1. Introduction

Maternal and fetal infections are important factors contributing to intrauterine fetal death (IUFD), stillbirth and preterm birth [1], making them one of the most challenging and devastating obstetric complications. Understanding the immunological mechanisms that underlie avoiding fetal wastage and clearing an infection might help to find optimal treatment conditions for pregnant women at risk. Thus far, the roles of neutrophils, macrophages and T cells in infection-induced fetal death have been studied, while the role of B cells is poorly defined.

Our understanding of the immune mechanisms that are activated during pregnancy has changed dramatically in recent years. Strong experimental evidence obtained from both animal models and patient samples suggests that both the mother and the fetus contribute to the symbiosis that is required to allow the pregnancy to come to term [2,3,4]. The maternal and fetal responses to infection, however, are quite different. A subclinical infection in the mother may jeopardize the well-being or even the survival of the fetus, as the maternal immune system aims to protect the mother at the detriment of the fetus. Conversely, upon the recognition of danger signals, the fetus may activate immune pathways to survive, which can involve the early onset of its own birth.

A negative correlation has been identified between the B cell number in maternal blood and birth weight in cases of intrauterine growth restriction [5]. We also reported that µMT mice that are deficient in mature B cells have pregnancies that come to term, but their fetuses are about 50% smaller at gd 7 than those of B cell-sufficient control animals [6]. Although these mice had normal T cell and Treg numbers, the Treg population did not expand during pregnancy, as is seen in normal control female mice. Contradicting the theory of increased susceptibility to infection in the presence of an increased Treg population, these mice could not expand their Treg pool but were much more susceptible to LPS than control mice [6]. These data implied that loss of mature B cells renders mice more susceptible to infections. The transfer of IL-10 producing B cells (B10) could prevent IUFD [6]. Consequently, our follow-up study aimed to understand the signaling mechanisms activated by LPS in maternal B cells and the role in pregnancy complications, particularly in IUFD. We also observed B10 expansion during pregnancy in mice and humans [7,8,9]: here, IL-10 helps to hamper the proinflammatory cytokines produced by maternal T cells [8]. The inability to expand this population is associated with fetal rejection in mice [7] and spontaneous abortion or preterm delivery in humans [8,9]. As such, transferring B10 cells or a subpopulation of B1a B cells characterized by high PC1 expression and IL-10 secretion can normalize the otherwise increased spontaneous abortion rate in an experimental mouse model [10]. B10 cells are often referred to as regulatory B cells (Bregs), being the extracellular markers B220^high^CD19^high^CD5^+^CD1d^high^ [11].

The gram-negative bacteria *Escherichia coli ssp.* is one of the most important pathogens contributing to stillbirth and IUFD [1,12,13,14]. The predominant immunologically active component of these bacteria is lipopolysaccharide (LPS), which is present in the outer cell membrane. LPS is predominantly recognized by toll-like receptor (TLR)-4 [15]: the signaling cascade that ensues can be divided into (i) a myeloid differentiation primary response gene 88 (MyD88)-dependent pathway and (ii) a TIR domain-containing adaptor inducing IFN-β (TRIF)-dependent pathway [16]. While both pathways activate NF-κB, the MyD88-dependent pathway induces proinflammatory cytokine secretion, while the TRIF-dependent pathway induces type 1 IFN production [16]. B cells also use another TLR, RP105 (CD180; Radioprotective 105 kDa protein), for LPS recognition. RP105, in turn, is regulated by CD19 [17].

We recently showed that while B cell-sufficient mothers and their fetuses do not succumb to a low LPS dose, fetuses from mothers lacking mature B cells (µMT mothers) undergo IUFD, while the pregnant females survive. The transfer of B10 cells could prevent fetal death, thus this particular IL-10 secreting B cell population seems to be a critical player in the immune balance that allows the coexistence of immunity against pathogens and tolerance towards the fetus [6]. The signaling pathway involved in this B cell-mediated response remained unclear. Here, we aimed to understand the participation of MyD88 herein, as it is the most important signal transducer for TLR4. For this, we generated mice in which MyD88 was specifically deleted from B cells (BMyD88^-/-^ mice) and employed CD19^-/-^ or MyD88^-/-^ mice as well as wild type counterparts as controls. We monitored the response to LPS exposure with the aim to understand how B cell-specific MyD88 signaling impacts LPS-driven infection in midgestation.

## 2. Materials and Methods

### 2.1. Animal Husbandry and Model Generation

C57BL/6 (H2^b^) WT littermates, CD19^cre/wt^ MyD88^flox/flox^ (B6.129P2(SJL)-*Myd88^tm1Defr^*/J) BMyD88^-/-^ mice, CD19^-/-^ mice lacking CD19 expression (B6.129P2(C)-^Cd19tm1(cre)Cgn^/J) and MyD88^-/-^ (B6.129P2(SJL)-*Myd88^tm1.1Defr^*/J) mice aged 8–12 weeks old were bred at the Medical University of Magdeburg. BALB/c (H2^d^) males were purchased from Janvier (Le Genest-Saint-Isle, France). All mice were housed on a 12 h light cycle. Chow and water were provided ad libitum.

Virgin female WT, BMyD88^-/-^, CD19^-/-^ and MyD88^-/-^ mice were mated with BALB/c males. Females were inspected twice a day for a vaginal plug. The presence of a vaginal plug was designated at day 0 of pregnancy. At day 10 of pregnancy (gd 10), 7–9 mice in the experimental group received a 200µL injection of LPS (3 µg/mL) into the peritoneal cavity, while the control group received 200µL PBS (N = 7–9 mice/group). Females were sacrificed 24 h after LPS administration (gd 11) and the spontaneous abortion rates within the groups were calculated.

### 2.2. Sample Collection and Histology

Blood was obtained by puncture of the retrobulbar vessel, 24 h after LPS administration. The blood sample was centrifuged at 4000× *g* for 10 min. at room temperature (RT). The serum was collected and stored at −80 °C until further use. One implantation site and one placenta per female was collected for paraffin embedding, 24 h after LPS challenge (gd 11). The implantation sites were fixed in 4% (*w/v*) paraformaldehyde (PFA) with 0.1 M sucrose (pH 7.4) for 6 h, and the placentas were fixed in 96% ethanol.

### 2.3. Quantitative Histological Measurements

Quantitative histological measurements of placental surface areas and whole implantation sites were performed on gd 11. Briefly, 5-μm transverse cross sections of feto–placental units were stained with H/E. AxioVision4 (Zeiss, Oberkochen, Germany) was used to measure the implantation size and placental surface areas at a 10x magnification.

### 2.4. B Cell Isolation and Stimulation

Splenic B cells were negatively isolated using a B cell Isolation Kit II (Miltenyi Biotech, Bergisch Gladbach, Germany) according to the manufacturer’s instructions. For short-term stimulation, 1 × 10^6^ isolated B cells/mL were cultured in the presence of Brefeldin A (control) or LPS (10µg/mL), PMA (5 ng/mL; both from Sigma; Munich, Germany), ionomycin (500 ng/mL; Thermo Fisher Scientific, Dreieich, Germany) and Brefeldin A (1×; Biolegend, San Diego, CA, USA) for 5 h at 37 °C and 5% CO_2_. Then, the B cells were analyzed by flow cytometry (see below). For long-term stimulation, 1 × 10^6^ isolated B cells/mL were cultured in the absence (medium control) or presence of LPS (10 µg/mL) for 24 h at 37 °C and 5% CO_2_. The supernatant was harvested, and cytokine levels were analyzed (see below).

Single-cell suspensions from decidua or inguinal lymph nodes (ILN) from gd 10 treated PBS- or LPS mice were obtained 24 h later as previously described [6]. Afterwards, cells were stimulated with PMA (5 ng/mL; both from Sigma; Munich, Germany), ionomycin (500 ng/mL; Thermo Fisher Scientific, Dreieich, Germany) and Brefeldin A (1×; Biolegend, San Diego, CA, USA) at 37 °C and 5% CO_2_ for 5 h.

### 2.5. Cell Staining and Flow Cytometry

Cultured B cells or cell suspensions from decidua and ILN were obtained and stained for cell surface markers for 30 min. at 4 °C. For the detection of intracellular markers, cells were fixed overnight using Fix and Perm (ebioscience, San Diego, CA, USA), and stained with the indicated antibodies (see Supplementary Material Table S1) for 30 min. at 4 °C. Measurements were performed on a Attune NxT (life technologies, Thermo Fisher Scientific, Darmstadt, Germany) or FACSCalibur (BD Biosciences, Heidelberg, Germany) and analyzed by FlowJo software (BD Biosciences, Ashland, OR, USA).

### 2.6. Cytokine Detection in Sera and Supernatants

IL-17A, TNF-α, IFN-γ, IL-2, IL-4, IL-10 and IL-6 levels in B-cell culture supernatants and serum samples were determined using a Mouse Cytometric Bead Array (CBA) Th1/Th2/Th17 Cytokine kit (BD Biosciences, Heidelberg, Germany) following the supplier’s recommendation.

### 2.7. Statistics

All statistical analyses were performed using GraphPad Prism 8.0 software (San Diego, CA, USA). The normality of data distributions was determined by the Shapiro–Wilk test. The obtained data among all experimental groups were evaluated by the nonparametric Kruskal–Wallis test, differences between two independent groups were calculated by the Mann–Whitney U test. Significance was defined as follows: * *p* < 0.05, ** *p* < 0.01, *** *p* < 0.001, **** *p* < 0.0001.

## 3. Results

### 3.1. MyD88-Deficient B Cells and CD19-Deficient B Cells from Pregnant Mice Have a Defective LPS Response

**Figure 1:**
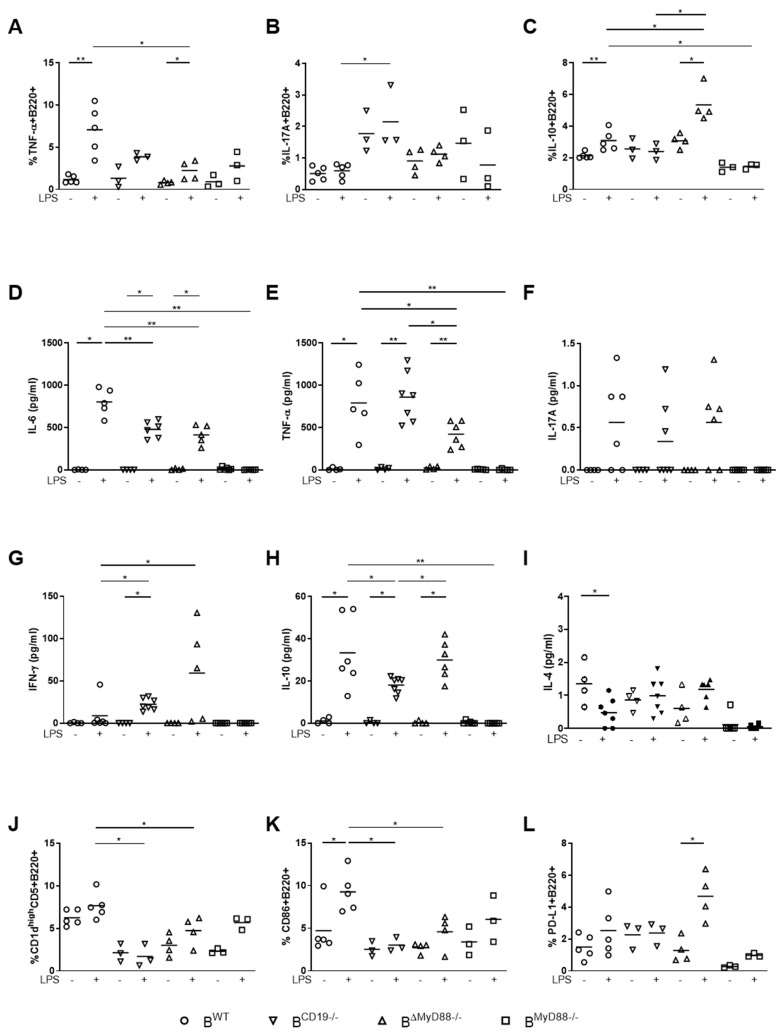
B-cell-specific MyD88 or CD19 knockout influences LPS responsiveness in vitro. Splenic B cells were isolated from pregnant WT (circle), BMyD88^-/-^ (triangle, point down), CD19^-/-^ (triangle, point upwards) and MyD88^-/-^ mice (square) at gestational day (gd) 10 and incubated with Brefeldin A (control) or Brefeldin A, LPS, PMA and ionomycin for 5 h. The frequencies of TNF-α^+^B220^+^ B cells (**A**), IL-17A^+^B220^+^ B cells (**B**) and IL-10^+^B220^+^ B cells (**C**) were analyzed by flow cytometry. The levels of IL-6 (**D**), TNF-α (**E**), IL-17A (**F**), IFN-γ (**G**), IL-10 (**H**) and IL-4 (**I**) produced by B cells from WT, BMyD88^-/-^, CD19^-/-^ or MyD88^-/-^ mice following 24 h of LPS stimulation or medium control incubation were determined by CBArray. The percentages of CD1d^high^CD5^+^B220^+^ B cells (**J**), CD86^+^B220^+^ B cells (**K**) and PD-L1^+^B220^+^ B cells (**L**) were determined by flow cytometry following 5 h incubation with either Brefeldin A (control) or Brefeldin A, LPS, PMA and ionomycin. The Kruskal–Wallis test and Mann–Whitney U test were used to analyze the data. N = 3–8 mice/group; * *p* < 0.05, ** *p* < 0.01. Shown are the mean values.

After first observations suggesting that MyD88 signaling in maternal B cells is relevant for fetal well-being [18], we aimed to determine whether the deficiency of B cell-specific MyD88 or CD19 expression influenced B cell subpopulations, in particular IgM + IgD^high^B220^+^ mature B cells, follicular (FO) B cells (CD1d^mid^CD5-CD21^mid^CD23^+^CD43-IgM^low^IgD^low/mid^B220^+^), marginal zone (MZ) B cells (CD1d^high^CD5-CD21^high^CD23-CD43-IgM^high^IgD^low^B220^+^), B1a B cells (CD1d^high^CD5^+^CD21^mid^CD23-CD43^+^IgM^high^IgD^low^B220^+^), B1b B cells (CD1d^mid^CD5-CD23-CD43^+^IgM^high^IgD^low^B220^+^) and Breg cells (CD1d^high^CD5^+^CD21^high^CD43-IgM^high^IgD^low^B220^+^) in blood, inguinal lymph nodes (ILN), paraaortic lymph nodes (PLN), spleen and in the peritoneal lavage. However, we detected no differences in the frequencies of these B cell populations despite a trend towards a higher frequency of Breg cells in WT mice (Supplementary Material Figure S1). Next, we analyzed the impact of LPS on B cell-secreted cytokines. We tested these mechanisms in B cells isolated from WT, CD19^-/-^ mice and BMyD88^-/-^ mice. B cells from MyD88^-/-^ mice served as additional controls. After mating the mentioned females allogeneically with male BALB/c mice, we sacrificed the mice on gd 10 and negatively sorted and stimulated splenic B cells with LPS, PMA and ionomycin for 5h to boost cytokine secretion in the presence of Brefeldin A for intracellular flow cytometry analysis (gating strategy show in Supplementary Material Figure S2). We found that 5 h of LPS stimulation boosted TNF-α secretion by WT B cells (Figure 1A). By contrast, only a small proportion of B cells from BMyD88^-/-^ and CD19^-/-^ mice produced this inflammatory cytokine (Figure 1A). The proportion of B220^+^ cells capable of produce IL-17A after LPS stimulation was augmented only in the CD19-deficient group that showed enhanced IL-17A secretion also without stimulation (Figure 1B). Interestingly, IL-10 production by isolated B cells from WT mice was augmented after LPS treatment and this was observed to an even greater extent in B cells from BMyD88^-/-^ mice; no changes were found for B cells from CD19^-/-^ and MyD88^-/-^ mice (Figure 1C). These findings demonstrate that deficiency of B-cell specific MyD88 or CD19 expression alters the LPS-induced cytokine secretion of B cells.

In our next set of experiments, we again isolated B cells and stimulated them with LPS for 24 h to investigate the profile of secreted cytokines by CBArray. The levels of IL-6 and TNF-α for cultured B cells from MyD88^-/-^ mice were very low and remained unchanged after LPS stimulation. Specifically, IL-6 expression was induced in B cells from WT, BMyD88^-/-^ and CD19^-/-^ mice after LPS stimulation, while the LPS-activated B cells from BMyD88^-/-^ and CD19^-/-^ mice produced less IL-6 than WT B cells (Figure 1D). While B cells from all groups, apart from MyD88^-/-^ mice, secreted more TNF-α following LPS stimulation than the medium controls (Figure 1E), B cells from BMyD88^-/-^ mice produced less TNF-α than those from WT mice and CD19^-/-^ mice. There was no detectable difference between B cells from WT and CD19^-/-^ mice (Figure 1E). IL-17A secretion was induced following LPS stimulation of B cells from WT, BMyD88^-/-^ and CD19^-/-^ mice, but not significantly (Figure 1F). For IFN-γ, we detected significantly higher levels of this cytokine produced in response to LPS in B cells isolated from CD19^-/-^ and the majority of BMyD88^-/-^ mice than from WT mice (Figure 1G). IL-10 was also upregulated following LPS stimulation in B cells derived from all mouse strains except from MyD88^-/-^ mice (Figure 1H). However, LPS-treated B cells from CD19^-/-^ mice produced significantly less IL-10 than WT and BMyD88^-/-^ B cells. IL-4 is essential for the activation of mature B cells as a cofactor for e.g., LPS, and induces B cell differentiation, proliferation, and antibody secretion, mainly IgG1 and IgE [19,20,21,22,23,24]. While its secretion is decreased following LPS stimulation in WT mice, it increased in BMyD88^-/-^ mice, but not significantly (*p* = 0.0601; Figure 1I). The secretion of all cytokines from B cells by MyD88^-/-^ mice was low and did not change following LPS stimulation. Taken together, these data show that loss of B cell specific MyD88 dampens the secretion of pro-inflammatory cytokines following LPS challenge with an induction of IFN-γ and IL-10, while loss of CD19 signaling limits the induction of the anti-inflammatory IL-10.

Next, we addressed the question which B-cell population might be responsible for the observed IL-10 release. Despite CD1d and CD5, regulatory B cell (Breg) populations might also be identified due to their expression of PD-L1 and CD86 [25,26]. It is known that PMA and ionomycin stimulation induces CD1d and CD5 expression by Breg cells [11]. Here we found that BMyD88^-/-^ and CD19^-/-^ mice had fewer CD1d^high^CD5+B220+ Bregs in response to LPS, PMA and ionomycin than WT mice (Figure 1J). An enhanced proportion of B cells from WT mice expressed the costimulatory molecule CD86 following 5 h of stimulation with LPS, PMA and ionomycin. While this trend was also found in the BMyD88^-/-^ group, but did not reach significance, no changes were observed in CD19^-/-^ mice (Figure 1K). A greater frequency of B cells from BMyD88^-/-^ mice expressed PD-L1, another Breg subset marker [27], after stimulation. This was not detected in B cells isolated from WT, CD19^-/-^ or MyD88^-/-^ mice (Figure 1L). These data indicate that, while WT mice predominantly bear Breg cells expressing CD1d^high^CD5^+^, B cell-specific loss of MyD88 favors the expression of PD-L1-expressing B cells.

### 3.2. Deficient B Cell-Specific MyD88 Signaling Hinders IUFD after LPS Treatment

**Figure 2:**
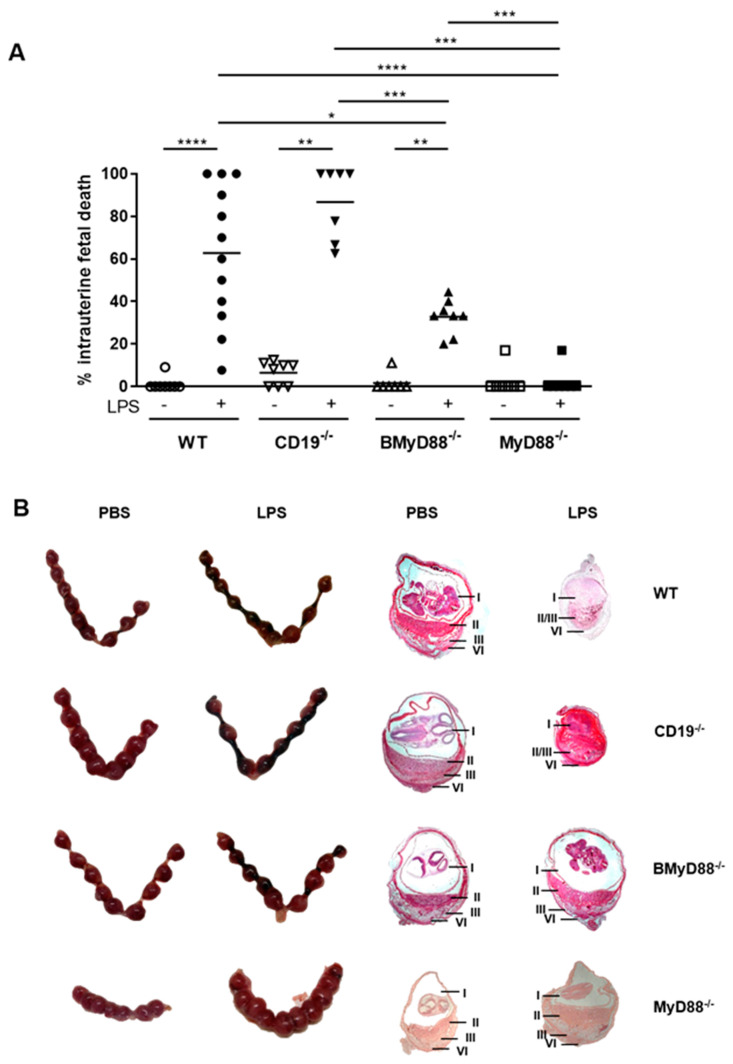
Fetuses from WT and CD19^-/-^ mice are more susceptible to LPS than those from BMyD88^-/-^ and MyD88^-/-^ mice. The rate of intrauterine fetal death was determined at 24 h after LPS or PBS injection in gestational day (gd) 10 pregnant WT, BMyD88^-/-^, CD19^-/-^ and MyD88^-/-^ mice. The Kruskal–Wallis test and Mann–Whitney U test were used to analyze the data; N = 7–9 mice/group; * *p* < 0.05, ** *p* < 0.01, *** *p* < 0.001, **** *p* < 0.0001 (**A**). Representative images captured of the uteri and whole implantation sites (**B**), showing I (amniotic cavity; II (placenta); III (decidua basalis) and IV (mesometrial lymphoid aggregate of pregnancy). Shown are the mean values.

As we recently reported, μMT mice lacking mature B cells are more susceptible to LPS-driven IUFD than WT mice [6]. Thus, we asked whether CD19^-/-^ mice have the same response and, if so, whether IUFD is driven by MyD88 signaling. At gd 10, we exposed females to 3 µg/mL LPS or PBS via intraperitoneal (i.p.) injection and determined the survival of the pups 24 h later. LPS exposure provoked IUFD in 63% of WT females and in 87% CD19^-/-^ females (Figure 2A). By comparison, LPS exposure resulted in IUFD in only 33% BMyD88^-/-^ females (*p* = 0.0319). MyD88^-/-^ females were, as expected, resistant to LPS, resulting in 2.5% IUFD, which was also detected in the PBS controls (Figure 2A). Hematoxylin and eosin (H/E) staining of whole implantation sites in CD19^-/-^ mice exposed to LPS showed that the architecture in the implantation was destroyed and, given that the identification of fetal or placenta structures were no longer possible (Figure 2B), the fetal structures surrounded by amniotic fluid, normally present in the amniotic cavity, could no longer be recognized; the layers of the placenta and decidua basalis were no longer distinguishable. This outcome was not observed in BMyD88^-/-^ and MyD88^-/-^ mice that presented clear fetal and placental structures. These data show that fetuses of pregnant mice lacking CD19 expression in B cells are more susceptible to LPS than WT controls. However, if MyD88 was specifically deleted from B cells, IUFD could be prevented.

### 3.3. Systemic Maternal Imbalance between Pro-Inflammatory and Anti-Inflammatory Processes Alters the Function of Decidual Tissue, Leading to IUFD

**Figure 3:**
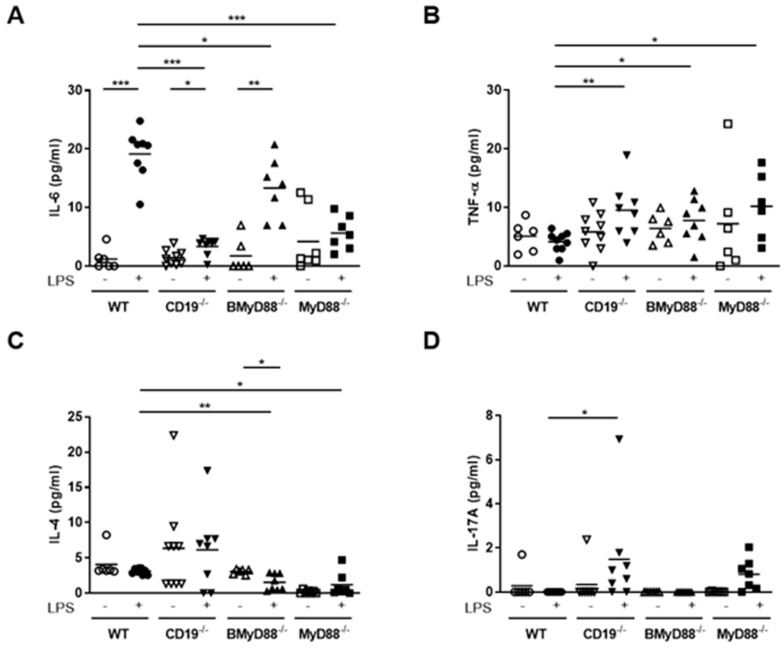
Cytokine levels were differently altered after in vivo LPS treatment. IL-6 (**A**), TNF-α (**B**), IL-4 (**C**) and IL-17A (**D**) levels in the serum of WT, BMyD88^-/-^, CD19^-/-^ and MyD88^-/-^ mice at 24 h after LPS or PBS injection on gestational day (gd) 10 were determined by CBArray. N = 7–9 mice/group; * *p* < 0.05, ** *p* < 0.01, *** *p* < 0.001. Shown are the mean values.

Upon inflammation, the maternal immune response has to resolve it while protecting the unborn at once. In order to determine how the B-cell specific alterations are reflected in the maternal systemic immune response, we studied the changes in cytokine concentrations in the serum of pregnant mothers exposed to LPS versus a sham (PBS) treatment, whose outcome was described in the preceding subsection. The concentration of IL-6 in the serum increased 24 h after LPS in vivo treatment compared to PBS injection in all mouse strains; however, the levels of secreted IL-6 in the serum of CD19^-/-^ mothers were lower than those in WT mothers at 24 h after LPS injection (Figure 3A). Compared to pregnant CD19^-/-^ females, BMyD88^-/-^ pregnant mothers showed an improved (higher) IL-6 response after LPS insult. IL-6 levels were, however, unchanged in MyD88^-/-^ controls after LPS treatment. The systemic level of TNF-α was also higher in LPS-treated BMyD88^-/-^ and CD19^-/-^ mice than in LPS-treated WT mice (Figure 3B). Furthermore, the loss of MyD88 in B cells resulted in diminished IL-4 secretion in response to LPS treatment (Figure 3C). IL-17A was detectable in CD19^-/-^ mice and MyD88^-/-^ controls following LPS treatment (Figure 3D).

**Figure 4:**
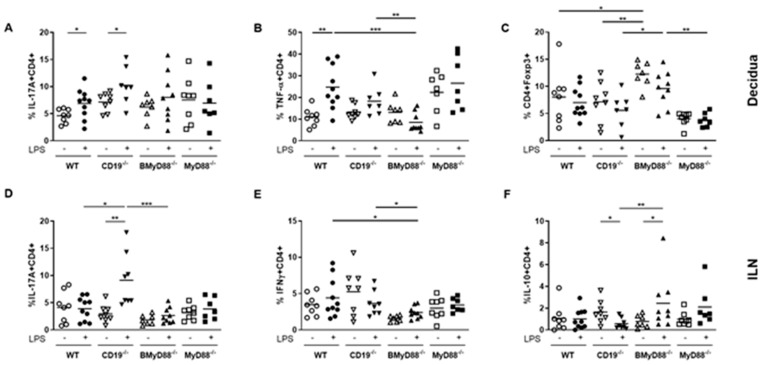
Loss of MyD88 or CD19 in B cells influences proinflammatory and regulatory T-cell population frequencies. Analysis of T cell populations in the decidua and inguinal lymph nodes (ILNs) of WT, BMyD88^-/-^, CD19^-/-^ and MyD88^-/-^ mice was performed at gestational day (gd) 11, 24 h after LPS or PBS injection. The frequencies of IL-17A^+^CD4^+^ T cells (**A**), TNF-α^+^CD4^+^ T cells (**B**) and CD4^+^Foxp3^+^ Tregs (**C**) in the decidua, and IL-17A^+^CD4^+^ T cells (**D**), IFN-γ^+^CD4^+^ T cells (**E**) and IL-10^+^CD4^+^T cells (**F**) in the ILNs were analyzed by flow cytometry. The Kruskal–Wallis test and Mann–Whitney U test were used to analyze the data. N = 7–9 mice/group; * *p* < 0.05, ** *p* < 0.01, *** *p* < 0.001. Shown are the mean values.

We next explored cytokine secretion by immune-cell subsets isolated from decidual part of the placenta of the mice with impaired B cell responses, used for the previous experiments (Figure 2) to gain an insight into the immune environment at the interface between fetus and mother. Here, we focused our analysis on T cells which were influenced by their interaction with B cells (flow cytometry gating strategy in Supplementary Material Figure S3). After 24 h in vivo LPS treatment, CD4^+^ T cells from WT and CD19^-/-^ mice produced more IL-17A compared to their corresponding PBS-treated controls, while no changes in IL-17A were observed in BMyD88^-/-^ or MyD88^-/-^ females (Figure 4A). Among the LPS-treated groups, no significant differences were detected. Within the decidua, CD4+ T cells from WT mice strongly induced TNF-α after 24 h LPS challenge compared to their PBS counterparts (Figure 4B). In addition, CD4^+^ T cells from LPS-treated CD19^-/-^ mice upregulated TNF-α, but not significantly. LPS-treated BMyD88^-/-^ mice showed diminished TNF-α production at 24 h compared to LPS-treated WT mice (Figure 4B). Compared to PBS-treated WT and CD19^-/-^ mice, PBS-treated BMyD88^-/-^ mice had increased Treg numbers in the decidua (Figure 4C). Following 24 h in vivo LPS challenge, we detected a loss of Tregs in WT, CD19^-/-^ and BMyD88^-/-^ mice, but the latter had still higher Treg numbers (Figure 4C). No alterations in the expression of TNF-α and the frequency of Treg cells were detected between PBS- and LPS-treated MyD88^-/-^ controls (Figure 4B, C and Supplementary Material Table S2).

We finally analyzed the ability of CD4+ T cells from the inguinal lymph nodes (ILNs) to secrete the proinflammatory cytokines IL-17A and IFN-γ and the anti-inflammatory cytokine IL-10. We did so to have a clear picture of the local changes in cytokine secretion by immune cells in secondary lymph organs. This is of extreme relevance in pregnancy settings as the peripheral immune response does not mirror the local changes as lymphocytes become activated in the draining lymph nodes [28]. We observed that CD4^+^ T cells from LPS-treated CD19^-/-^ animals secreted more IL-17A than those from PBS-treated controls but found no differences among the other groups (Figure 4D). Interestingly, in the same mice, in vivo LPS treatment diminished the ability of CD4^+^ T cells to secrete IFN-γ (Figure 4E). For the anti-inflammatory cytokine IL-10, CD4^+^ T cells from LPS-treated CD19^-/-^ mice secreted reduced amounts of IL-10 compared to WT, which reflects the high IUFD rate. Finally, in BMyD88^-/-^ mice, CD4^+^ T cells secreted increased amounts of IL-10 if previously treated with LPS (Figure 4F and Supplementary Material Table S2). These data indicate that B cell specific alterations in the expression of either MyD88 or CD19 also influence the T cell specific immune response as a consequence of LPS challenge. While BMyD88^-/-^ mice favor a Treg/IL-10 response, WT and CD19^-/-^ mice induce a shift towards pro-inflammatory T cells.

Taken together, our results show that the protection of IUFD in mice with selective MyD88 deletion in B cells after LPS treatment goes in hand with a better ability to keep the balance between a protective anti-inflammatory and a potentially harmful pro-inflammatory immune response in the mother and the feto-maternal interface as well. This in turn indicates that MyD88 is the activated signaling pathway in B cell response to LPS application during pregnancy.

## 4. Discussion

Clinical and subclinical infections during pregnancy are associated with serious obstetrics complications, including intrauterine fetal death (IUFD). Although neglected for many years [29], it is now known that B cells are important for pregnancy success because of the multiple and pleiotropic functions they take over. Besides secreting antibodies that are relevant during pregnancy (AAbs), they are crucial regulators of T cell activity [30]. We also reported that µMT mice that are deficient in mature B cells, have pregnancies that come to term, but their fetuses are smaller than those of B cell-sufficient control animals [6].

After observing that µMT mice are more susceptible to LPS if pregnant, we aimed here to understand the signaling mechanisms activated by LPS in maternal B cells and the role in IUFD. To achieve this, we first studied pregnancy outcomes and the response to LPS in CD19^-/-^ mice that lack CD19 expression in B cells. Similar to µMT mice, these mice had a high susceptibility to LPS. To understand the participation of MyD88 in maternal B cell signaling, we studied how B cells react to LPS stimulation. We chose MyD88 because it has different functions depending on the cell type, which suggests that it can direct the immune response in opposite directions. For example, in epithelial cells, MyD88 signaling can regulate cell proliferation and stimulate the secretion of antimicrobial peptides [31,32], while in dendritic cells and macrophages MyD88 signaling regulates the secretion of proinflammatory cytokines [33,34,35]. We observed that a deficiency in MyD88 specifically in B cells resulted in fetal protection after maternal LPS exposure during pregnancy. This finding indicates that maternal B cell intrinsic MyD88 signaling is responsible for activating the proinflammatory mechanisms that can protect the mother but kill the fetus.

From our previous studies we concluded that B10 cells exert an important function in pregnancy by regulating Treg numbers [6]. CD19^-/-^ mice lack marginal zone (MZ) B cells, B1 cells and CD1d^high^CD5^+^ B10 cells and have decreased Treg numbers [36,37,38]; the transfer of B10 cells has been shown to increase Treg numbers [39,40]. The current findings support these data by showing that the frequency of CD4^+^Foxp3^+^ Tregs in the decidua and the number of IL-10-expressing CD4^+^ T cells in the lymph nodes of CD19^-/-^ mice was lower than the number detected at the same sites of BMyD88^-/-^ females after LPS challenge. LPS stimulation was not sufficient to induce a CD1d^high^CD5+ B-cell phenotype *in vitro*. We did not determine severely reduced numbers of MZ B cells and B1 B cells in gd 10 pregnant CD19^-/-^ mice; whether this is due to hormonal regulation or caused by the allogenic mating and the resulting microchimerism remains to be further investigated. CD19 regulates RP105-mediated signal transduction, and CD19^-/-^ B cells proliferate less than WT B cells following LPS stimulation [17]. In our model of LPS-induced IUFD, we observed that fetal survival in CD19^-/-^ mice was diminished compared to the fetal survival rate seen in BMyD88^-/-^ mice. B cells from CD19^-/-^ mice secreted more IL-17A and IFN-γ but failed to produce protective anti-inflammatory cytokines such as IL-4 or IL-10 as B cells from WT or BMyD88^-/-^ mice did. Moreover, the frequency of IL-17A-producing CD4^+^ T cells was also increased in CD19^-/-^ mice 24 h after LPS challenge. Both cytokines, IL-17A and IFN-γ, were shown to be involved in spontaneous abortion in humans and rodent models [41,42,43]. Enhanced IL-17A level were accompanied by reduced Treg cell numbers [44,45] and could be lowered e.g., by therapeutic treatment with intravenous immunoglobulin [46], indicating that full-competent B cells contribute to the Treg/Th17 ratio. These data suggest that the fetuses from these mice succumb to an excessive inflammation upon LPS.

We found that B cells from CD19^-/-^ mice at gd 10 did not upregulate costimulatory molecule CD86 expression. Previous studies have shown that signaling via the TLR RP105 induces CD86 expression [47,48,49]. In humans with one or two defective CD19 alleles, TLR stimulation induces CD19 phosphorylation via MyD88 but fails to upregulate the expression of CD86 and the transmembrane activator and CAML interactor (TACI), another important B cell molecule [50]. The combination of an anti-RP105 antibody with LPS-activated B cells induces rapid polyclonal Ig production via a T cell and MyD88-independent pathway [47].

Next, we aimed to understand the molecular pathways underlying B cell-mediated LPS-driven IUFD. For this, we concentrated on MyD88, the most important signal transducer for TLR4. Upon LPS stimulation, MyD88-dependent pathways (e.g., IL-6) and MyD88- and TRIF-dependent pathways (e.g., TNF-α) are activated. However, these cells produced increased IFN-γ levels upon LPS treatment. It has already been described that B cell-intrinsic MyD88 signals induce IFN-γ production by T cells [51]. B cells obtained from MyD88^-/-^ mice that we included here as controls, produced neither pro- nor anti-inflammatory cytokines following LPS stimulation, which supports previous reports that these mice show LPS unresponsiveness.

LPS stimulation can boost IL-10 production by B cells [48]. B cell-intrinsic MyD88 induces IL-10-producing CD19^+^CD138^+^ B cells, while MyD88 deficiency is associated with an increased number of B1 cells [52], which are important IL-10 producers [53]. Here, we showed that IL-10 production was upregulated by B cells from BMyD88^-/-^ mice at gd 10. While the frequencies of CD1d^high^CD5^+^ B10 cells and CD86^+^B220^+^ cells were unaltered following LPS treatment, PD-L1 expression was induced. PD-L1 is another molecule implicated in Breg function [27] and might contribute to IL-10 secretion by LPS-activated B cells. One important function of B cell-derived IL-10 is to decrease the release of TNF-α and IL-6 by other immune cells, such as macrophages [53]. Indeed, in the decidua, we found that TNF-α release by CD4^+^ T cells was reduced in BMyD88^-/-^ mice; this effect correlated with an enhanced frequency of Tregs. Moreover, in the ILNs of LPS-treated BMyD88^-/-^ mice, we detected that CD4^+^ T cells secreted less IL-17A and IFN-γ but more IL-10 than those in the ILNs of WT CD19^-/-^ mice.

Overall, we found that in female mice with MyD88-deficient B cells, the inflammatory immune response to LPS treatment is dampened by the induction of anti-inflammatory IL-10-mediated immunity. In this way, inflammation is resolved, while feto-maternal tolerance is maintained. CD19^-/-^ mice, however, failed to control the LPS-induced inflammation. This highlights the importance of a fine-tuned immune response during pregnancy, also in the event of inflammation. This is also important in pregnant patients since a major challenge in obstetrics is to identify pregnant women with an enhanced risk to develop severe complications during infections such as preterm delivery. Infections might also damage the placenta, leading to hypoxia and undersupply with essential nutrients, and finally to fetal death.

Another group of patients at higher risk for stillbirth are women with a history of fetal loss [54]. This might be caused, at least partly, by an imbalanced immune response due to an inadequate switch to a tolerogenic, IL-10 and TGF-β- mediated immunity. Combined with an infection that would otherwise be cleared without affecting the unborn, this might result in an overwhelming pro-inflammatory response, damaging the fetus. Thereby, these women should be closely examined for the presence of infections or alterations in the number of immune cells to treat them in time to avoid fetal death. Another high-risk group are women with small-for-gestational-age (SGA) babies. In these patients, it was shown that peripheral maternal lymphocytes produced increased levels of pro-inflammatory cytokines such as TNF-α and IL-6, but decreased IL-10 [55,56], combined with increased B cell numbers [5].

It has to be stated that our study has some limitations that had to be considered: The frequency of B lymphocytes in the decidua at gd 10 is too low to allow any analysis in more detail. Therefore, we cannot provide further information about B cell subpopulation(s) in gestational tissues that might be involved in IUFD. Furthermore, we cannot exclude that another molecule, e.g., a yet undefined cytokine, which acts downstream of B cell-specific MyD88 signaling, is either directly or indirectly important for the regulation of IUFD. However, we provide clear evidence of relevant B cell specific pathways involved in LPS-induced inflammation in a pregnancy model. This information is relevant for future studies aimed at designing strategies for preventing intrauterine fetal death, following Gram negative infections.

## Data Availability

The datasets in this study are available from the corresponding author upon reasonable request.

## Supplementary Materials

The following are available online at https://www.mdpi.com/article/10.3390/cells10102693/s1, Table S1: Antibodies used in this study; Table S2: T cell populations in decidua and ILN; Figure S1: Mature B cell populations in B cell-modified mice; Figure S2: Gating of B cell populations; Figure S3: Gating of T cell populations.
