# Supplementary material for: Maternal B Cell-Intrinsic MyD88 Signaling Mediates LPS-Driven Intrauterine Fetal Death
Source: Cells. 2021 Oct 8;10(10):2693. doi: 10.3390/cells10102693 (PMC8534512; doi:10.3390/cells10102693)
Supplement: Supplementary file 1 [file cells-10-02693-s001.zip › cells-1371743-supplementary.pdf]

Supplementary Material

Table S1. This table shows the antibodies used in the study.

Antigen	Conjugation	Isotype	clone	Manufacturer
		Extracellular		
B220	BV605	Rat IgG2a, κ	RA3-6B2	BD Biosciences
CD1d	PerCP-Cy5.5	Rat IgG2b, κ	1B1	BD Biosciences
CD4	FITC	Rat IgG2a, κ	RM4-5	BD Biosciences
CD5	BV421	Rat IgG2a, κ	53-7.3	BD Biosciences
CD86	FITC	Rat IgG2a, κ	GL1	BD Biosciences
PD-L1	BV421	Rat IgG2a, λ	MIH5	BD Biosciences
CD21/35	PE-Cy7	Rat IgG2a, λ	eBio8D9 (8D9)	eBioscience
CD22.2	BV711	Rat IgG1, κ	Cy34.1	BD Biosciences
CD23	BV510	Rat IgG2a, κ	B3B4	BD Biosciences
IgM	PE-CF594	Rat IgG2a, κ	R6-60.2	BD Biosciences
IgD	APC-H7	Rat IgG1	11-26c.2a	BD Biosciences
CD43	FITC	Rat IgG2a, κ	S7	BD Biosciences
		Intracellular		
Foxp3	AlexaFluor660	Rat IgG2a, κ	FJK-16s	eBioscience
IFN-γ	BV421	Rat IgG1, κ	XMG1.2	BD Biosciences
IL-10	PE	Rat IgG2b	JES5-16E3	BD Biosciences
IL-10	APC	Rat IgG2b	JES5-16E3	BD Biosciences
IL-17A	PE	Rat IgG1, κ	TC11-18H19	BD Biosciences
IL-17A	PE-Cy7	Rat IgG2a, κ	eBio17B7	eBioscience
TNF-α	PE	Rat IgG1	MP6-XT22	BD Biosciences
TNF-α	PerCP-Cy5.5	Rat IgG1	MP6-XT22	BD Biosciences

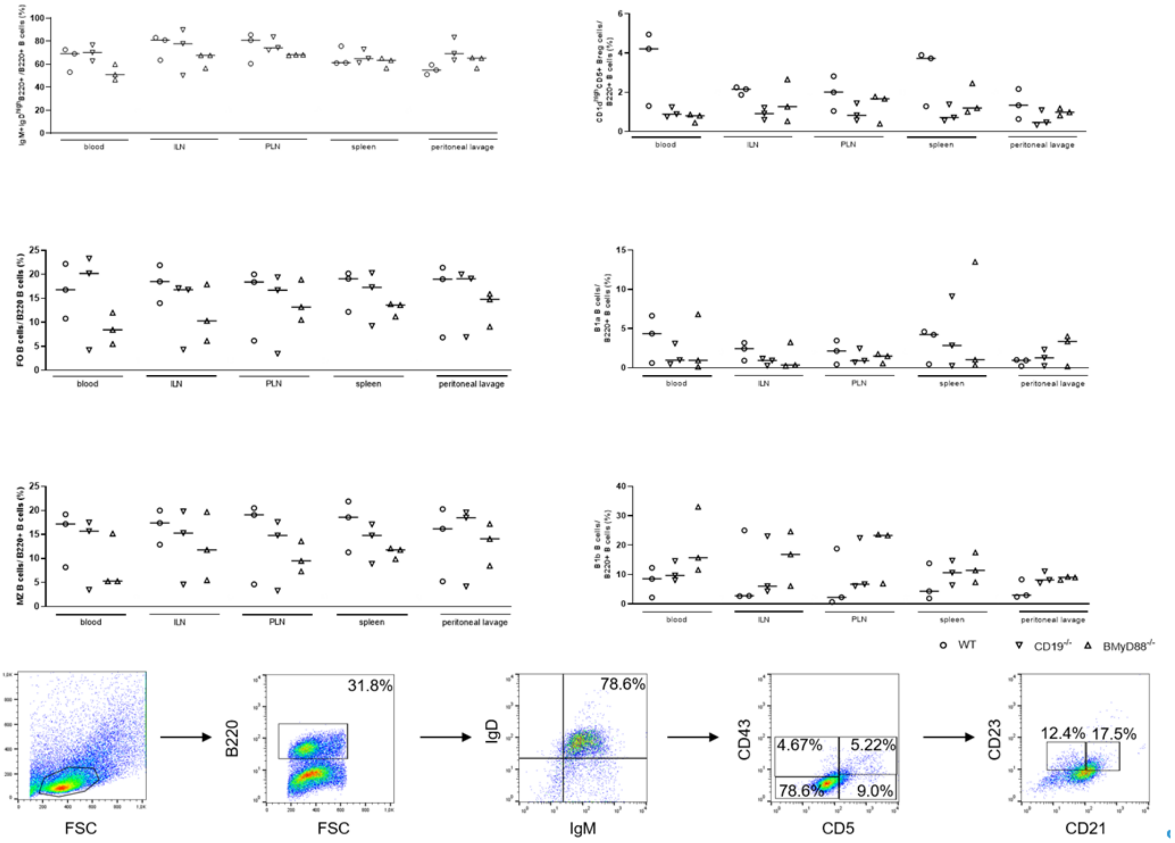

Figure S1. Mature B cell populations in B cell-modified mouse strains included in the study. IgM+IgDhighB220+ mature B cells, follicular (FO) B cells, marginal zone (MZ) B cells, B1a B cells, B1b B cells and Breg cells were detected and analyzed by flow cytometry in blood, inguinal lymph nodes (ILN), paraaortic lymph nodes (PLN), spleen and in the peritoneal lavage. Mean values are depicted in the graphic without standard deviation for the sake of clarity. No statistically significant differences were observed among the groups. The gating strategy is shown below: following gating lymphocytes, B220+ B cells and then IgM vs. IgD- expressing B cells were gated. The following gating included CD43 vs. CD5 staining, allowing to differentiate B1a, B1b, Breg and B2 cells. Cells were further gated upon CD21 vs. CD23 to differentiate FO vs. MZ B cells.

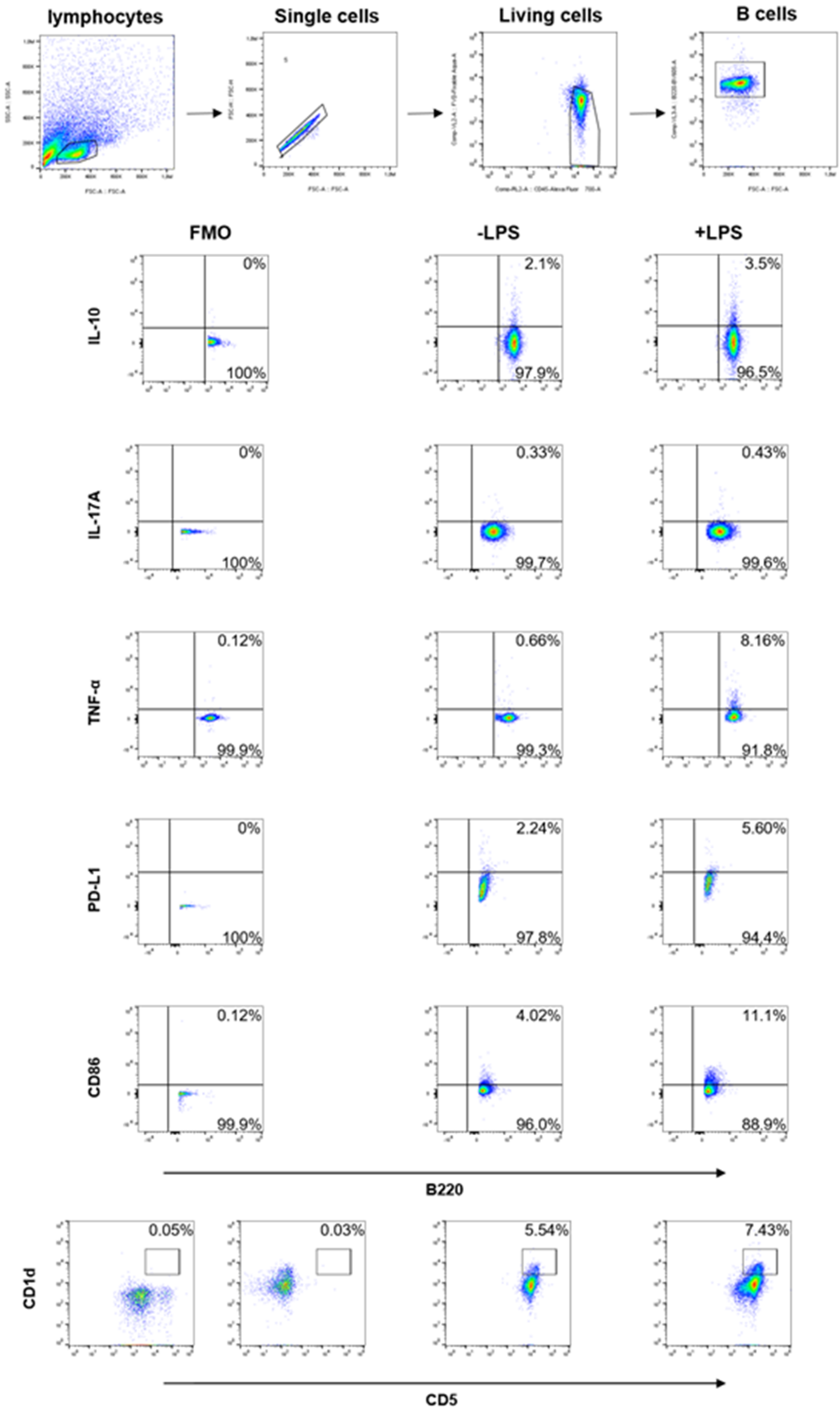
lymphocytes
Single cells
Living cells
B cells
FMO
-LPS
+LPS
IL-10
0%
100%
2.1%
97.9%
3.5%
96.5%
IL-17A
0%
100%
0.33%
99.7%
0.43%
99.6%
TNF-α
0.12%
99.9%
0.66%
99.3%
8.16%
91.8%
PD-L1
0%
100%
2.24%
97.8%
5.60%
94.4%
CD86
0.12%
99.9%
4.02%
96.0%
11.1%
88.9%
B220
CD1d
0.05%
0.03%
5.54%
7.43%
CD5

Figure S2. Gating of B cell populations; Following gating lymphocytes, single cells and living cells were identified, then the B220+ B cell gate was set. Positive stainings for IL-10, IL-17A, TNF-α, PD-L1, CD86 and CD1d/CD5 was identified using FMO controls. Here, we show exemplary results obtained following 5 h culturing of splenic WT B cells with either Brefeldin A (-LPS) or LPS, PMA, ionomycin and Brefeldin A (+LPS).

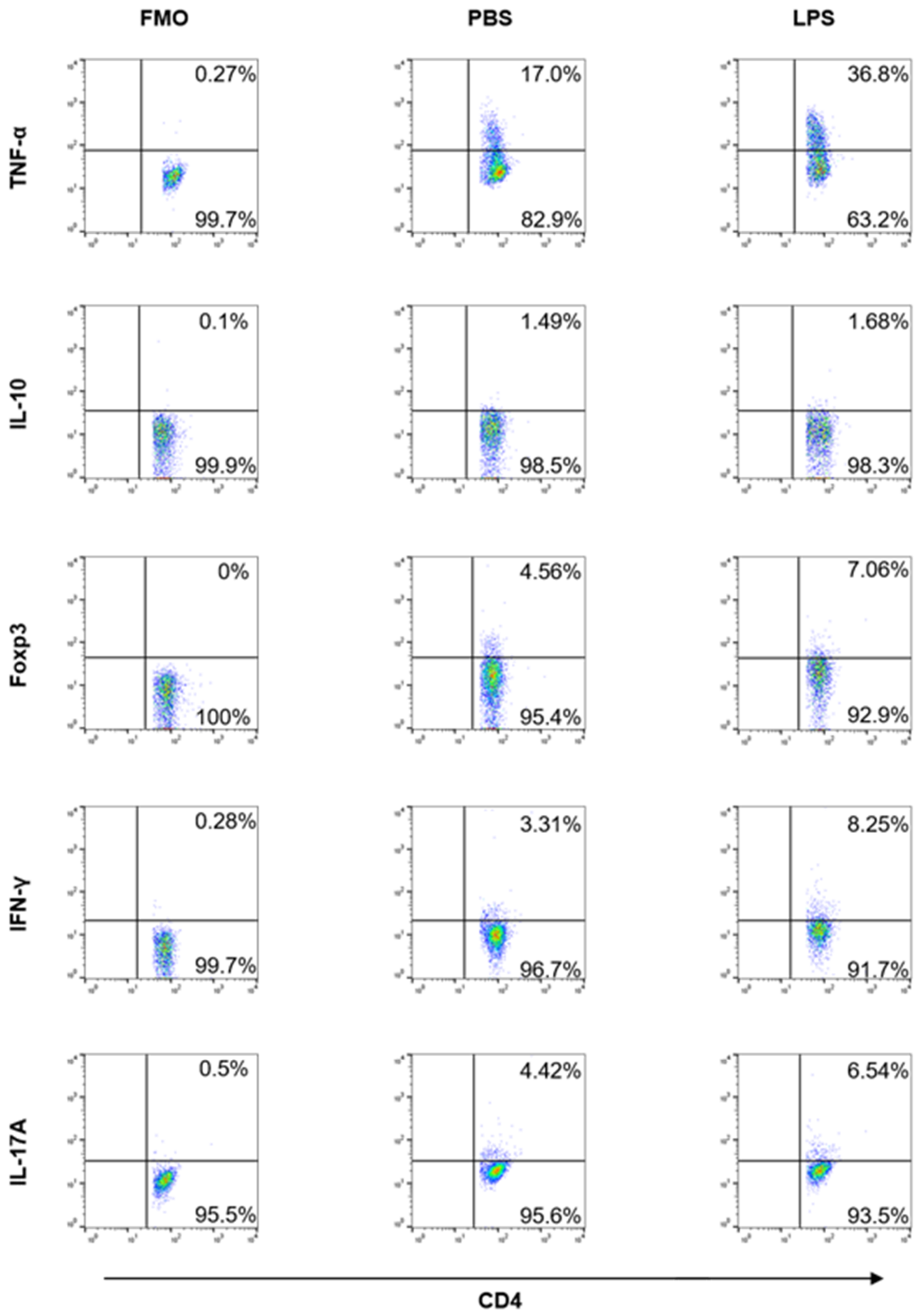

Figure S3. Gating of T cell populations. Following the gating lymphocytes, a gate was set for CD4+ T cells. Positive stainings for Foxp3, IL-10, IFN-γ, TNF-α and IL-17A were identified and defined using FMO controls. Single-cell suspensions from decidua or inguinal lymph nodes (ILN) were obtained 24 h after injection of PBS or LPS at gd 10 from PBS control mice (PBS) or LPS-treated mice (LPS). Cells were cultured with PMA, ionomycin and Brefeldin A, shown are exemplary results from WT PBS and WT LPS females.

Table S2. T cell populations in decidua and ILN.

	**WT**		**BMyD88$^{-/-}$**		**CD19$^{-/-}$**		**MyD88$^{-/-}$**	
LPS	**-**	**+**	**-**	**+**	**-**	**+**	**-**	**+**
Decidua								
TNF-α^+CD4$^+$ (%)	11.0	24.8	13.1	8.5	13.3	18.3	22.3	26.6
IFN-γ^+CD4$^+$ (%)	23.2	28.2	26.4	19.5	20.3	23.3	7.2	7.8
IL-17A$^+$CD4$^+$ (%)	4.6	6.9	6.2	8.0	7.2	10.2	7.5	7.0
CD4$^+$Foxp3$^+$ (%)	8.0	7.0	12.3	9.6	7.1	5.6	4.0	3.8
IL-10$^+$CD4$^+$ (%)	4.6	4.4	6.9	9.0	5.6	3.4	4.6	6.2
ILN								
TNF-α^+CD4$^+$ (%)	29.3	26.3	24.5	25.2	19.5	38.7	36.5	38.0
IFN-γ^+CD4$^+$ (%)	3.5	4.4	1.6	2.4	5.2	3.6	3.0	3.4
IL-17A$^+$CD4$^+$ (%)	4.1	3.9	1.8	2.7	3.0	9.1	3.2	3.9
CD4$^+$Foxp3$^+$ (%)	3.5	3.9	4.0	3.8	7.1	3.7	6.2	5.3
IL-10$^+$CD4$^+$ (%)	1.1	1.0	0.8	2.4	1.6	0.6	1.0	2.1

Table S2 shows mean values for proinflammatory and regulatory T-cell population frequencies of WT, BMyD88$^{-/-}$, CD19$^{-/-}$ and MyD88$^{-/-}$ mice in decidua and ILN at gd 11, 24 h after LPS or PBS injection. The values are shown as mean. We refrained of including S.D. for the sake of clarity.
